# Host- and genotype-specific transcriptomic responses to *Mycoplasma bovis* infection in bison and Holstein peripheral blood mononuclear cells

**DOI:** 10.3389/fimmu.2026.1873273

**Published:** 2026-08-06

**Authors:** Bryan S. Kaplan, Kaitlyn Sarlo Davila, Jamison R. Slate, Steven C. Olsen, Rohana P. Dassanayake, David J. Holthausen, Daniel W. Nielsen, Randy E. Sacco

**Affiliations:** 1 Ruminant Diseases and Immunology Research Unit, National Animal Disease Center, Agricultural Research Service, United States Department of Agriculture, Ames, IA, United States; 2 Oak Ridge Institute for Science and Education, Agricultural Research Service (ARS) Research Participation Program, Oak Ridge, TN, United States; 3 Infectious Bacterial Diseases of Livestock Research Unit, National Animal Disease Center, Agricultural Research Service, United States Department of Agriculture, Ames, IA, United States

**Keywords:** bison, Holstein, *Mycoplasma bovis*, peripheral blood mononuclear cells, transcriptomics

## Abstract

The bacterial pathogen *Mycoplasma bovis* (*M. bovis*) emerged as the etiologic agent of mastitis in cattle during the early 1960’s. Concurrent with the emergence of *M. bovis*, Holsteins in the US were undergoing intensive genetic selection for increased milk production. Comparison studies with unselected Holsteins have found an association with selection and increased susceptibility to disease. More recently, *M. bovis* was identified as the cause of epizootics of severe, chronic respiratory disease in American bison (*Bison bison*). In cattle, *M. bovis* causes chronic respiratory disease and is associated with the bovine respiratory disease complex while in bison, *M. bovis* is considered a primary pathogen. The objective of this study was to characterize and compare the transcriptomic response of peripheral blood mononuclear cells (PBMCs) from contemporary Holsteins (CH), unselected Holsteins (UH), and bison at 24 hr following *in vitro M. bovis* infection. The greatest and fewest number of differentially expressed genes (DEG) were observed in UH and bison, respectively. Ingenuity pathway analysis of DEG identified the genotype-specific expression of pathways involved in cytokine and chemokine response, neutrophil degranulation, and antigen processing and presentation. This study is the first to characterize how PBMC transcriptomic responses to *in vitro M. bovis* infection differ by genotype (CH vs. UH) and species (Holstein vs. bison). These findings provide insight into observed differences in clinical disease between bison and cattle and highlight the effects of primary selection for milk production on Holstein responses to *M. bovis*.

## Introduction

*Mycoplasma bovis* (*M. bovis*) is a small, wall-less bacterium that is a significant pathogen of multiple ruminant species including cattle (*Bos taurus*) and bison (*Bison bison*). First identified in 1961 from a severe outbreak of mastitis in a dairy herd (1), *M. bovis* is now endemic in most major cattle-producing countries worldwide (2, 3). The first cases of *M. bovis* infection in bison were reported in 1999 (4) and *M. bovis* is now recognized as a significant threat to bison ranching and restoration (5). Despite both ruminant species being susceptible to *M. bovis* infection, distinct differences in pathogenesis have been observed. In cattle, *M. bovis* is frequently associated with the multifactorial bovine respiratory disease (BRD) complex (6) causing pneumonia, arthritis, and otitis media in calves and yearlings and mastitis in dairy cows. Conversely, *M. bovis* is recognized as a primary pathogen in bison (7, 8), frequently associated with high mortality epizootics of chronic respiratory disease in managed herds. In bison, *M. bovis* is reported to cause a plethora of clinical syndromes including polyarthritis, pharyngitis, laryngitis, pleuritis, pericarditis, and reproductive disorders. Further, adult animals frequently present with clinical disease and recent studies show asymptomatic infection of bison calves is common (9). Genomic analysis of cattle and bison *M. bovis* isolates did not identify specific virulence factors or mutations that would impact the contrasting disease phenotypes observed in each ruminant species (10), which suggests that species-specific differences in the host response underlie the divergent etiology, pathogenesis, and clinical disease syndromes.

Concurrent with the emergence of *M. bovis* as a pathogen of cattle in the 1960’s, Holsteins in the US were undergoing intensive genetic selection for increased milk production (11). The resulting contemporary Holstein (CH) produces greater milk yields than the previously unselected Holstein (UH) population; however, this genetic selection has altered immune, metabolic, and endocrine functions that are hypothesized to have detrimental effects on animal health and reproduction (12, 13). In 1964 the University of Minnesota established an unselected control line, now known as UH, by breeding cows to sires of average milk production of the mid 1960’s (14, 15). Genomic analyses comparing the CH to the UH identified changes in approximately 40% of the genome between the genetic lines, with significant changes in genomic loci associated with genes involved with immunity (13, 16). The functional effects of this selection have been demonstrated by experimental challenge studies that found increased immune function in UH as compared to CH (17, 18). Following intravenous lipopolysaccharide administration, UH produce increased proinflammatory cytokines such as tumor necrosis factor alpha (TNF-α) and interleukin-6 (IL-6) levels and had reduced milk bacterial counts following intramammary challenge with *Escherichia coli* (18). Further, stimulation of whole blood with heat-killed bacteria from UH resulted in greater production of IL-1β and IL-6 (19).

To further investigate the ruminant immune response and characterize the effects of host genomic background on *M. bovis* infection, we compared the transcriptomes of *M. bovis* infected peripheral blood mononuclear cells (PBMC) isolated from CH, UH, and bison by RNA sequencing (RNAseq). Our data demonstrated that PBMC from both genotypes of Holsteins have a greater transcriptional response than bison to *M. bovis* infection. The transcriptional responses of both UH and bison PBMC to *M. bovis* infection were characterized by increased expression of genes involved in Th1 immunity and proinflammatory signaling pathways. Our studies demonstrated that the transcriptional responses of *M. bovis* infected PBMC differs by species and between Holsteins genotypes.

## Materials and methods

### Animals

Seven lactating, primiparous contemporary and unselected Holstein cows were housed and managed under the same conditions at the National Animal Disease Center dairy facility. Cows were all between two and three years of age and between 63 and 152 days in milk. Five female bison, approximately two years of age, were housed at the National Animal Disease Center in Ames, IA. All procedures were evaluated and approved by the National Animal Disease Center Animal Care and Use Committee.

### Isolation of bovine and bison peripheral blood mononuclear cells

Holstein and bison PBMC were isolated from whole blood collected via jugular venipuncture into syringes containing 2x acid citrate dextrose buffer. Blood was diluted 1:2 in Dulbecco’s phosphate buffered saline (DPBS) (ThermoFisher, Waltham, MA, USA) and layered onto pre-loaded Sepmate tubes (Stem Cell Technologies, Vancouver, British Columbia, Canada) containing 15 ml of Lymphoprep (Stem Cell Technologies). Filled Sepmate tubes were centrifuged for 15 minutes at 1,200 x g at room temperature. The top plasma layer was then removed and the buffy coat transferred into a clean 50 ml conical tube. Recovered cells were washed in DPBS and pelleted by centrifugation at 300 x g for 10 minutes at room temperature. Ammonium chloride potassium lysis (ACK) buffer was used to remove contaminating red blood cells. Cells were again washed in DPBS and pelleted by centrifugation at 300 x g for 10 minutes. PBMC were filtered through a 40 μM strainer, pelleted by centrifugation at 300 x g for 10 minutes, and resuspended in complete RPMI 140 (cRPMI) (ThermoFisher) containing 10% heat-inactivated fetal bovine serum (FBS), 2 nM L-glutamine, 1% essential amino acids, 1% non-essential amino acids, 1% sodium pyruvate, antibiotic/antimycotic solution (ThermoFisher), and 50 μM 2-mercaptoethanol (Sigma Aldrich, Burlington, MA). Cell counts and viability were obtained by using a ViCell Blue cytometer (Beckman Coulter, USA) according to manufacturer’s instructions. The volume of PBMC suspension was adjusted to obtain a final concentration of 10^7^ cells/ml.

### Mycoplasma bovis

The *M. bovis* isolate NADC1 was used for infection of PBMC. Axenic cultures of NADC1 were grown in in PPLO broth (BD Diagnostic Systems, Franklin Lakes, NJ, USA) supplemented with 10g/L of yeast extract (BD Diagnostic Systems), and 20% horse serum (v/v) (ThermoFisher) for 48 hrs at 37 C, 5% CO_2_ and aliquots were stored at -80 C. Prior to infection, a frozen aliquot of NADC1 suspension was thawed and cultured in 3 ml PPLO broth at 37 C, 5% CO_2_ for 20 hrs. The *M. bovis* inoculum was quantified by performing serial ten-fold dilution in DPBS and plating 5 µl in triplicate on PPLO agar plates. The plates were incubated at 37 C, 5% CO_2_ for 72 hr prior to enumeration.

### Infection of PBMC and RNA isolation

For *in vitro* experiments, 100 μl of PBMC suspension from each of the 7 Holsteins/genotype and each of the 5 bison were used to seed wells of a 96-well plate (1x10^6^ cells/well). The NADC1 inoculum was prepared by pelleting bacterial cells, from 3 ml PPLO cultures, by centrifugation at 15,000 x g for 20 min. The pellet was resuspended in cRPMI media and 100 μl of inoculum was used to infect PBMC at a multiplicity of infection (MOI) of approximately 0.5. An additional 100 μl of cRPMI was added to mock infected wells. Cells were incubated for 24 hrs at 37 C, 5% CO_2_. Following the incubation period, PBMC were pelleted by centrifugation at 400 x g for 5 minutes at 4 C. The supernatants were removed and the cells were lysed by adding 100 μl/well TRIzol reagent (ThermoFisher) and incubated at room temperature for 5 min. Cell lysates were transferred to clean 0.2 ml tubes and stored at -80 C. Purification of RNA was performed using the Purelink RNA Mini kit (ThermoFisher) according to manufacturer’s instructions.

### RNA sequencing and differential gene expression analysis

Library preparation was performed using the Illumina Stranded mRNA Library Preparation Kit and Sequenced on a P2 flow cell on the NextSeq 1000 (Illumina Inc., San Diego, CA, USA) to generate 100-bp paired end reads with an average of 22.06 million reads per sample. RNA samples from each of the 19 animals used in this study were not pooled prior to use in library preparation. Tools at galaxy.scinet.usda.gov were utilized to analyze the sequenced reads. Quality control checks were performed with FastQC v0.12.0. TrimGalore v0.6.5 was used to remove adapters and reads with a phred score below 20 prior to alignment on the Bos Taurus ARS-UCD 2.0 or Bison UMD 1.0 genome assemblies, respectively, with HISAT2 v2.1.0 (20). Raw counts were generated with FeatureCounts v.2.0.1 (21) and differential gene expression (DEG) was performed using DeSeq2 v2.11.40.6 (22). DEG were considered significant if log_2_FC > |1| and Benjamini-Hochberg False Discovery Rate < 0.05. Ingenuity Pathway Analysis (IPA) by Qiagen was used to determine canonical pathways associated with the differentially expressed genes (DEG) in each host type. Raw sequence data is available on GenBank (PRJNA 1439942).

## Results

### Differential gene expression in *M. bovis* infected CH, UH, and bison PBMC

**Figure 1:**
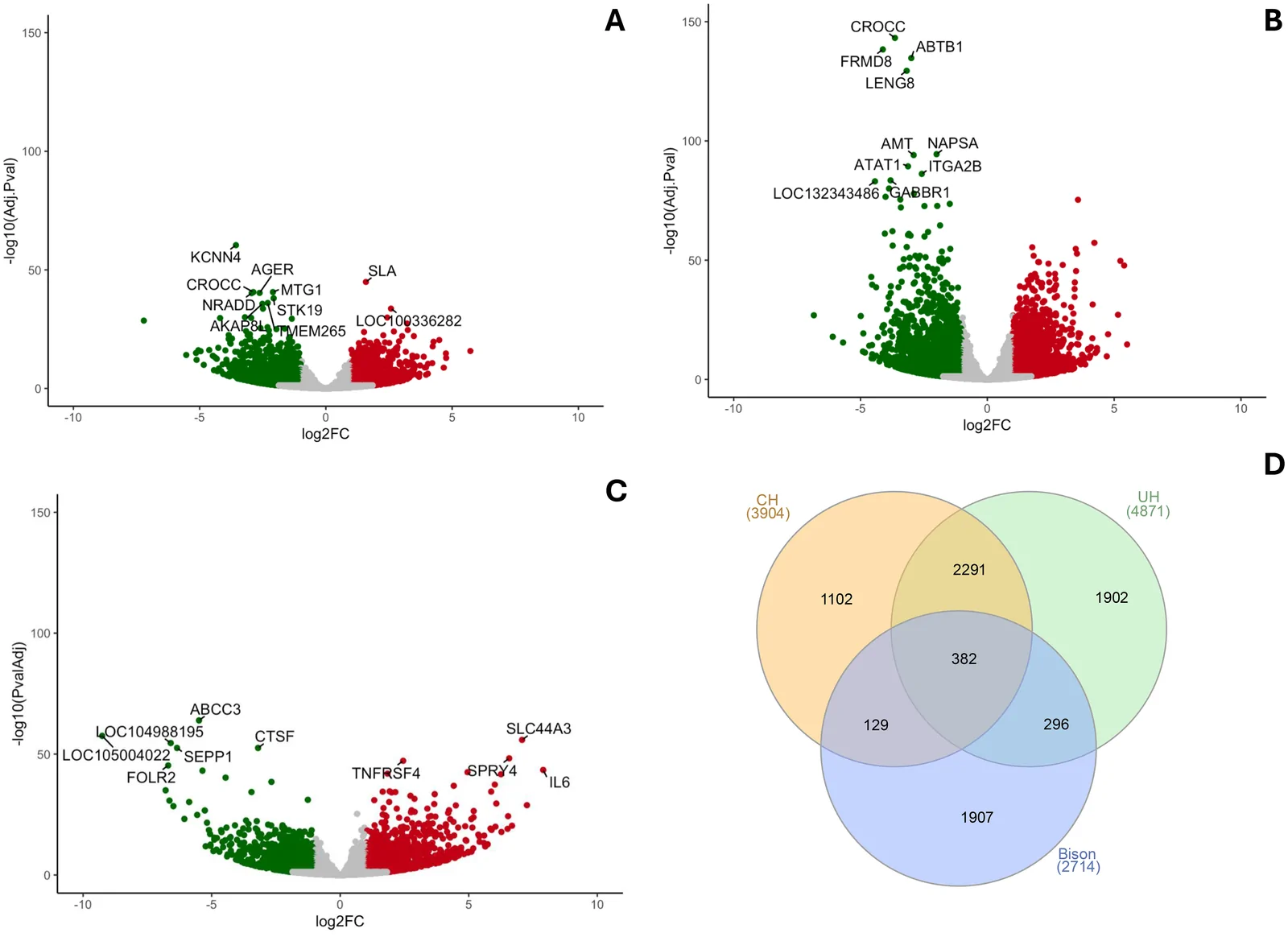
*Mycoplasma bovis* induced differential gene expression in PBMC from contemporary Holsteins, unselected Holsteins, and bison. Volcano plots show differential gene expression in contemporary Holsteins **(A)**, unselected Holsteins **(B)**, and bison **(C)**. Significant genes (Benjamini–Hochberg FDR *p* < 0.05) downregulated are shown in green, while upregulated genes are shown in red. Genes that are not significantly differentially expressed are shown in grey. The number of DEGs shared between genotypes is plotted in the Venn diagram **(D)**.

To compare the responses to *M. bovis* infection of each cattle genotype and bison, RNAseq was performed on PBMC 24 hrs post-infection. Volcano plots showing the number of upregulated and downregulated DEG were generated for CH (**Figure 1A**), UH (**Figure 1B**), and bison (**Figure 1C**). The number of significant DEG (P < 0.05) in *M. bovis* infected PBMC compared to uninfected PBMC in contemporary Holsteins (CH), unselected Holsteins (UH), and bison were 3904, 4871, 2714, respectively. Of the 3904 significant DEG in CH, 1651 were upregulated while 2253 were downregulated (**Supplementary Table 1**). The greatest number of DEG were observed in *M. bovis* infected UH PBMC where out of a total of 4871 DEG, 2018 DEG were upregulated while 2853 DEG were downregulated (**Supplementary Table 2**). The lowest number of DEG were observed in *M. bovis* infected bison PBMC where out of a total of 2714 DEG, 1212 DEG were upregulated and 1502 DEG were downregulated (**Supplementary Table 3**). As expected, the transcriptomic response of PBMC to *M. bovis* infection was more similar between Holstein genetic lines. CH and UH shared 2291 common DEG where only 129 CH DEG and 296 UH DEG overlapped with bison (**Figure 1D**). Interestingly, 382 DEG were common between all three genotypes. A list of all unique and shared DEG by genotype is provided in **Supplementary Table 4**.

In CH, the two most significant DEG were *SLA*, encoding for Src-like adaptor protein, involved in the negative regulation of T-cell receptor signaling (23), and *KCNN4*, encoding for the Potassium Calcium-Activated Channel Subfamily N, Member 4, is important for the formation of multinucleate giant cells (24). Others include *CROCC* (Ciliary Rootlet Coiled-Coil), *MTG1* (Mitochondrial Ribosome Associated GTPase1), *AGER* (Advanced Glycosylation End-Product Specific Receptor), and *NRADD* (Neurotrophin Receptor Associated Death Domain) (**Supplementary Table 1**). The most significant DEG in UH were *CROCC*, *FRMD8* (FERM Domain-Containing Protein 8), *ABTB2* (Ankyrin Repeat and BTB/POZ Domain-Containing Protein 2), and *LENG8* (Leukocyte Receptor Cluster Member 8) (**Supplementary Table 2**) all of which were downregulated. In bison PBMC, *ABCC3* (ATP Binding Cassette Subfamily C Member 3), *LOC1050040*, *SLC44A3* (Solute Carrier Family 44 Member 3), and *LOC1049881* were the most significant DEG following *M. bovis* infection (**Supplementary Table 3**).

### Comparative pathway enrichment in *M. bovis* infected CH, UH, and bison PBMC

**Figure 2:**
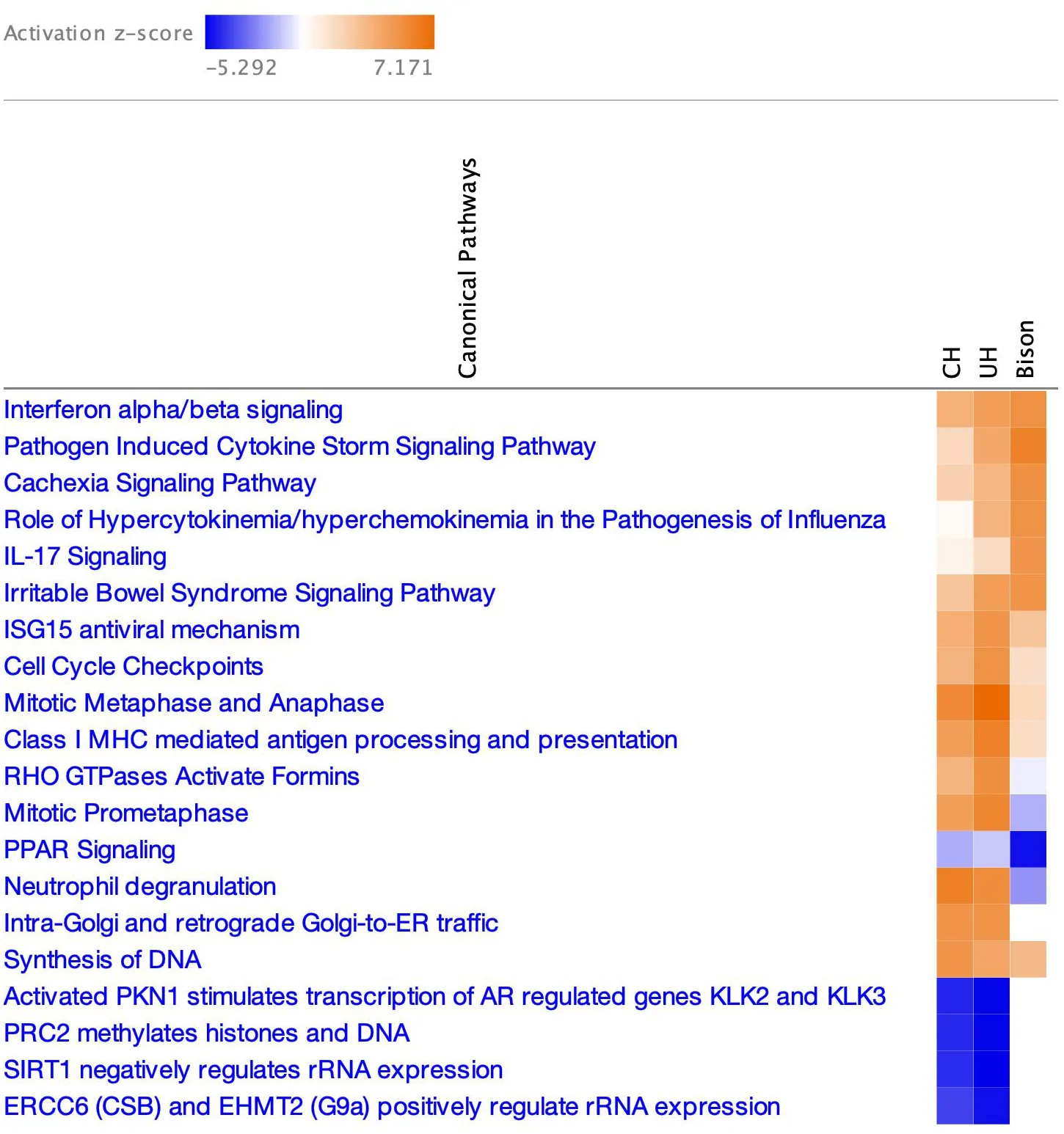
Heat map of the top pathways across all three genotypes based on p value and z score are shown. Positive z scores indicating pathway activation are shown in orange while negative z scores indicating pathway inhibition are shown in blue. Greater shading indicates a greater magnitude of activation or inhibition.

Analysis of significantly enriched pathways further revealed the divergent transcriptional responses between Holstein genetic lines and bison PBMC. A total of 529, 589, and 266 pathways were significantly enriched (P < 0.05) in CH (**Supplementary Table 5**), UH (**Supplementary Table 6**), and bison (**Supplementary Table 7**) PBMC, respectively. Enriched pathways with increased expression in *M. bovis* infected CH PBMC were primarily involved with innate immune cell function and signaling including neutrophil degranulation while those with decreased expression were involved in wound healing, IL-17A signaling, and osteoarthritis pathways (**Figure 2**; **Supplementary Table 5**). Enriched pathways observed in *M. bovis* infected UH PBMC were similar in CH however differences in expression level were observed between the two genotypes. The neutrophil degranulation pathway had slightly reduced expression levels in UH as compared to CH while pathways involved in the adaptive immune response against intracellular pathogens (class I MHC mediated antigen processing and presentation, bacterial pathogen pattern recognition receptor signaling) and cytokine signaling (interferon signaling, pathogen induced cytokine storm signaling) were upregulated in UH as compared to CH (**Figure 2**; **Supplementary Table 6**). In contrast to both Holstein genotypes, the transcriptome of *M. bovis* infected bison PBMC was characterized by significantly enriched pathways involved with cytokine and chemokine signaling and classical macrophage activation and a decrease in the neutrophil degranulation pathway (**Figure 2**; **Supplementary Table 7**). The bison PBMC response had the greatest expression of genes comprising the signaling pathways for interferon α/β, pathogen induced cytokine storm, IL-17, cachexia, and the macrophage classical activation.

### Species-specific differences in the upregulation of the pathogen induced cytokine storm signaling pathway

**Figure 3:**
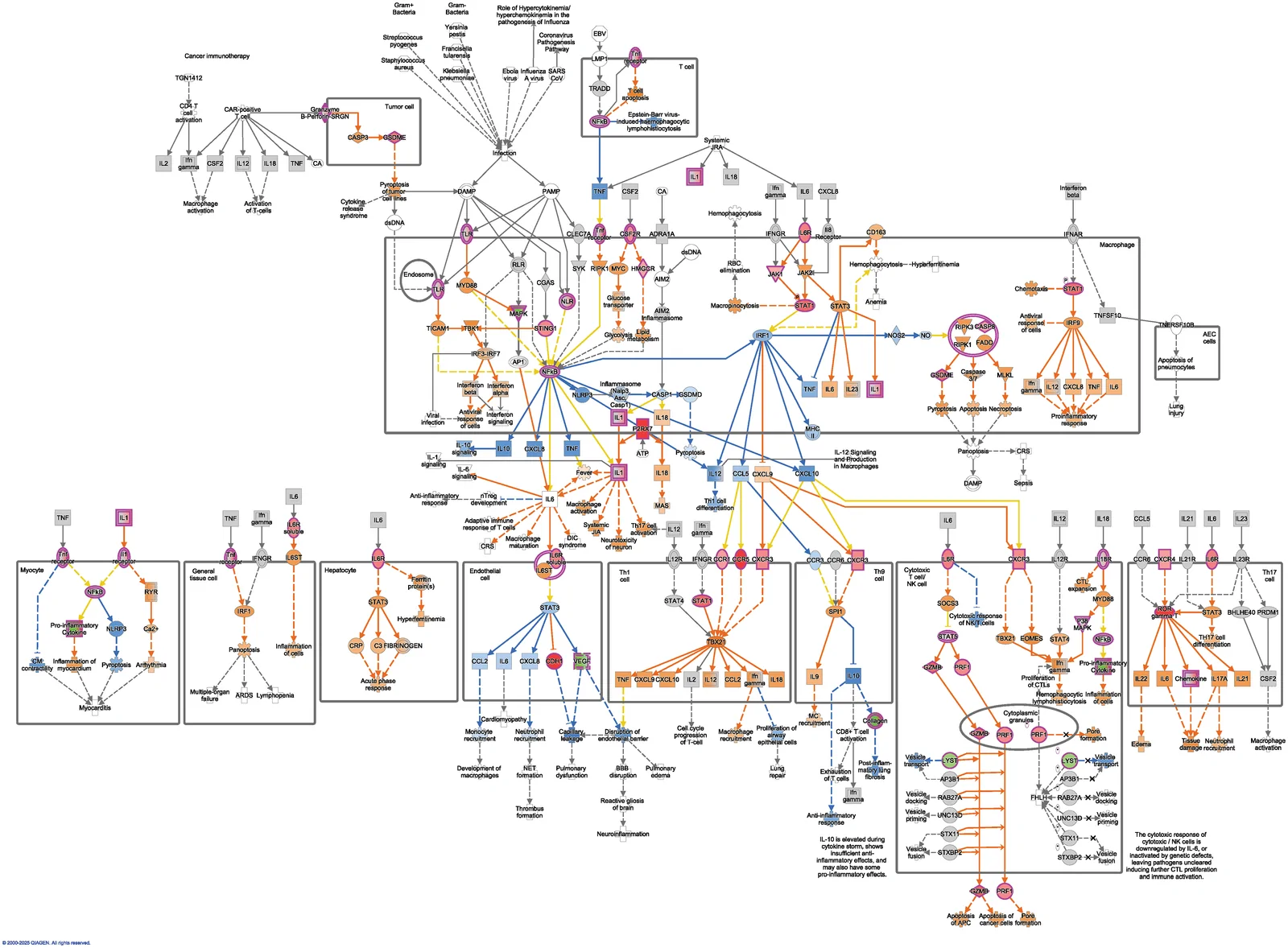
Differential gene expression post *Mycoplasma bovis* infection in contemporary Holstein PBMCs within the canonical IPA pathway “Pathogen Induced Cytokine Storm Signaling Pathway”. Downregulated genes are shown with green fill, while upregulated genes are shown with pink fill. The greater the upregulation or downregulation, the darker the fill. A molecule activity predictor tool predicts downstream activity based on significant differential gene expression. Predicted activation is shown in orange, and predicted inhibition is shown in blue. The more confident the prediction, the darker the fill. Solid lines represent direct relationships, while dashed lines represent indirect relationships.

**Figure 4:**
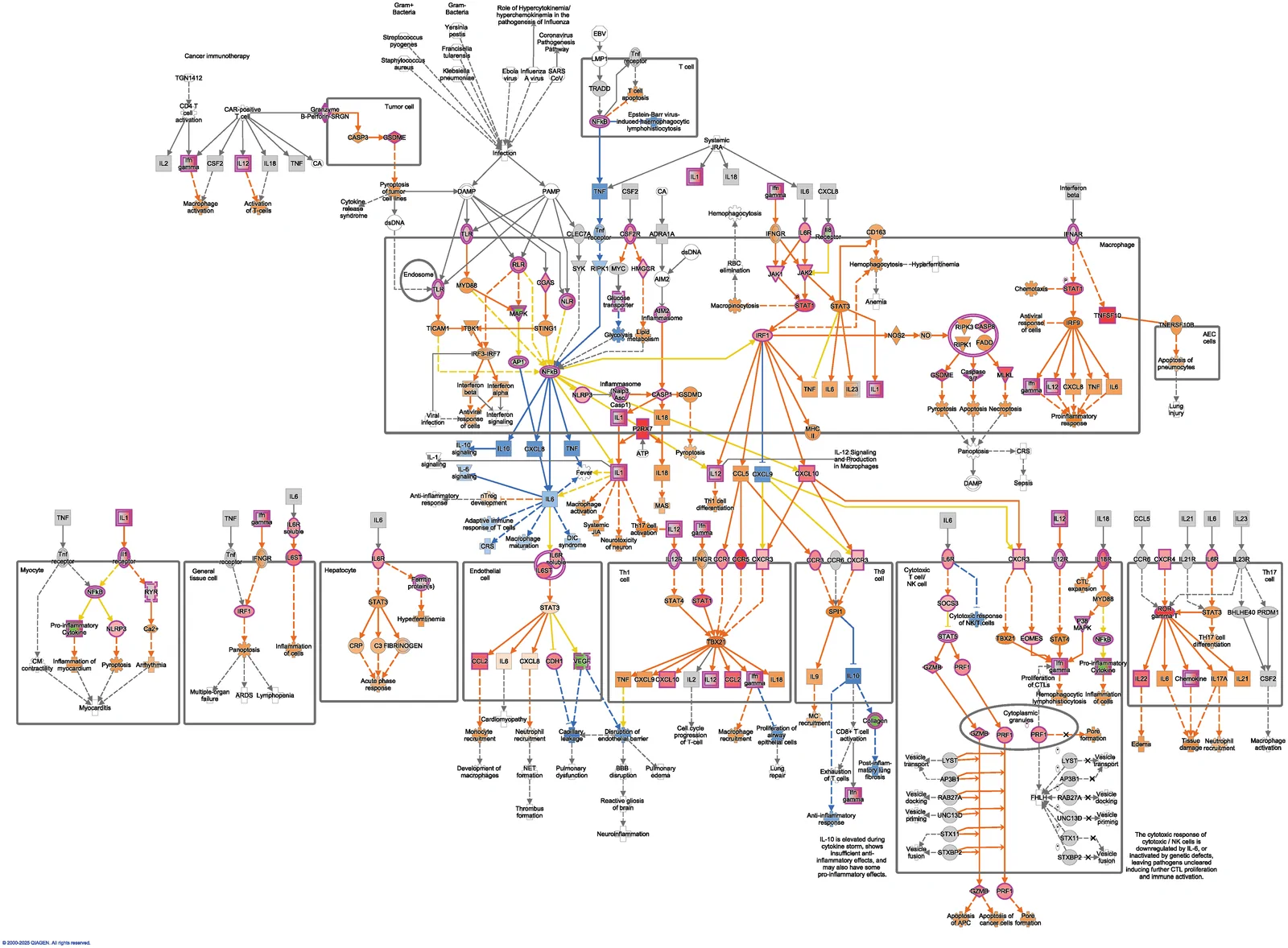
Differential gene expression post *Mycoplasma bovis* infection in unselected Holstein PBMCs within the canonical IPA pathway “Pathogen Induced Cytokine Storm Signaling Pathway”. Downregulated genes are shown with green fill, while upregulated genes are shown with pink fill. The greater the upregulation or downregulation, the darker the fill. A molecule activity predictor tool predicts downstream activity based on significant differential gene expression. Predicted activation is shown in orange, and predicted inhibition is shown in blue. The more confident the prediction, the darker the fill. Solid lines represent direct relationships, while dashed lines represent indirect relationships.

**Figure 5:**
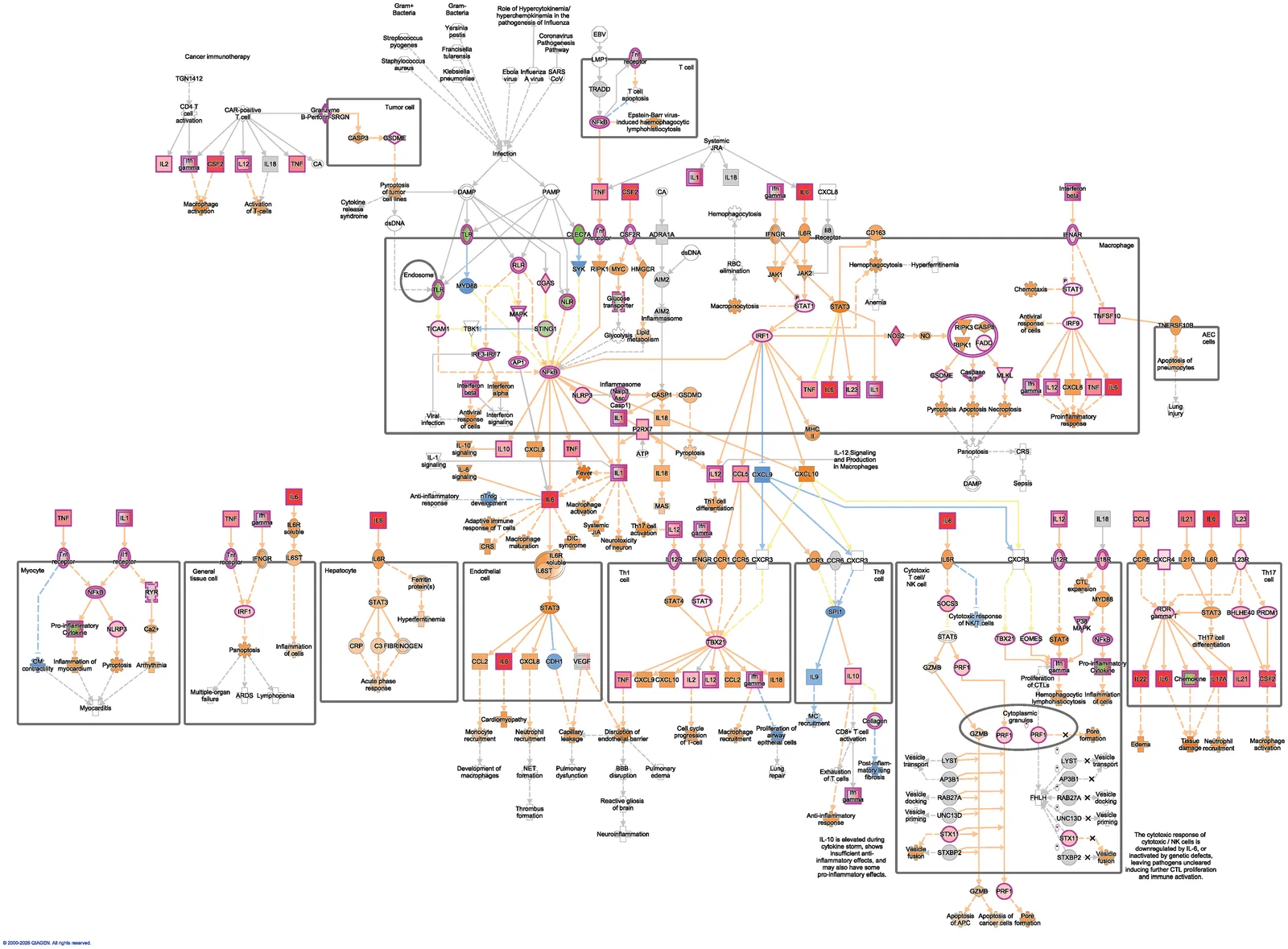
Differential gene expression post *Mycoplasma bovis* infection in bison PBMC within the canonical IPA pathway “Pathogen Induced Cytokine Storm Signaling Pathway”. Downregulated genes are shown with green fill, while upregulated genes are shown with pink fill. The greater the upregulation or downregulation, the darker the fill. A molecule activity predictor tool predicts downstream activity based on significant differential gene expression. Predicted activation is shown in orange, and predicted inhibition is shown in blue. The more confident the prediction, the darker the fill. Solid lines represent direct relationships, while dashed lines represent indirect relationships.

The pathogen induced cytokine storm signaling pathway (PICSS) showed increasing DEG by genetic lineage following *M. bovis* infection of CH (**Figure 3**), UH (**Figure 4**), and bison (**Figure 5**) PBMC and species-specific differences in DEG comprising the PICSS primarily encode for immune signaling molecules, their receptors, and transcription factors. Bison PBMC had the greatest number of significantly upregulated interferon, cytokine, and chemokine genes reflecting broad activation of immune signaling. In bison PBMC, type I and type II interferons, *IFNB1* (1.95 log_2_FC) and *IFNG* (4.23 log_2_FC), were upregulated while only *IFNG* was observed to be upregulated in UH (3.86 log_2_FC). Interestingly, only bison PBMC displayed an increase in *TNF* (3.28 log_2_FC), encoding the pro-inflammatory signaling molecule TNF-α which is consistent with the activation of the cachexia pathway (**Figure 2**). Compared to Holsteins, bison PBMC (**Figure 5**) showed increased expression of multiple cytokines including *IL1A* (5.87 log_2_FC), *IL2* (2.03 log_2_FC), *IL6* (7.9 log_2_FC), *IL10* (2.42 log_2_FC), *IL17A* (6.51 log_2_FC), *IL21* (3.93 log_2_FC), and *IL23A* (3.04 log_2_FC). Increased expression of *IL12A* and *IL22* was observed in both UH and bison PBMC, although bison PBMC demonstrated greater expression of *IL12A* and *IL22* (3.48 and 6.27 log_2_FC) as compared to UH (2.04 and 2.1 log_2_FC). Genes encoding chemokines involved in macrophage recruitment, *CCL2* (2.23 log_2_FC) and *CXCL10* (1.94 log_2_FC), were upregulated in UH while CCL5 was only upregulated in bison (3.08 log_2_FC).

Upregulation of chemokine receptors was most pronounced in Holsteins. CH showed increased expression of *CCR1* (1.6 log_2_FC), *CCR5* (2.64 log_2_FC), *CXCR3* (1.46 log_2_FC), and *IL6R* (1.6 log_2_FC) (**Figure 3**). *CCR1* (1.65 log_2_FC), *CCR5* (3.25 log_2_FC), *CXCR3* (1.03 log_2_FC), *IL6R* (1.5 log_2_FC), *IL12RB2* (3.27 log_2_FC), and *IL18R1* (2.03 log_2_FC) were upregulated in UH PBMC, while *CXCR1* (-1.72 log_2_FC) and *CXCR2* (-1.05 log_2_FC) were downregulated (**Figure 4**). DEG chemokine receptors in *M. bovis* infected bison PBMC included *IL18R1* (2.16 log_2_FC) and *IL23R* (1.08 log_2_FC). Interestingly, the expression of *CXCR4* was increased in CH (1.21 log_2_FC), UH (1.38 log_2_FC), and bison (1.1 log_2_FC) PBMC following *M. bovis* infection.

The differential expression of transcription factors critical for development of inflammatory and T cell responses were also influenced by *M. bovis* infection in ruminant PBMC. All three genotypes CH, UH, and bison demonstrated increased expression of *RORC*, the gene that encodes RORγt, which is critical for differentiation of Th17 cells. Bison PBMC (**Figure 5**; **Supplementary Table 3**) also increased *TBX21* expression (1.84 log_2_FC). In both Holstein genotypes, the transcription factor *STAT1* was upregulated though expression was higher in UH (2.54 log_2_FC) compared to CH (1.93 log_2_FC). In contrast, both Holstein groups demonstrated significant downmodulation of *NFκB2* (-1.06 log_2_FC), encoding the NFκB p100 subunit (CH: -1.06 log_2_FC, UH: -1.12 log_2_FC) whereas the gene was upregulated in bison PBMC (2.2 log_2_FC). Surprisingly, increased expression of SOCS3 was observed in UH (1.07 log_2_FC) and bison (2.13 log_2_FC).

*M. bovis* infection of ruminant PBMC increased expression of genes involved in apoptosis, necroptosis, and pyroptosis. Increased expression of *CASP8* (CH: 1.25 log_2_FC, UH: 1.9 log_2_FC) and *GSDME* (CH:1.84 log_2_FC, UH: 2.62 log_2_FC), involved in apoptosis, were observed in both genotypes of Holsteins. Conversely, *TNFSF10*, critical for apoptosis, and *MLKL*, involved in necroptosis, were both upregulated in UH and bison (**Supplementary Tables 2**, **S3**). Bison also had increased expression of NLRP3 (1.82 log_2_FC), a central component of the inflammasome involved in pyroptosis, for which differences in expression were not noted in Holstein PBMC.

## Discussion

This analysis demonstrates that cattle and bison PBMCs have fundamentally different transcriptomic responses to *M. bovis* infection. In our analysis, Holstein PBMC had the greatest transcriptomic response to *M. bovis* infection with the largest number of DEG being observed in UH PBMC. Our data suggest that following *M. bovis* infection of CH in Holstein PBMC, the greatest upregulation occurred in SLA, encoding for the Src-like adaptor protein (SLAP) that when expressed in T cells interacts with the T cell receptor and inhibits the activation of the transcription factor AP-1, which is important for cytokine expression (25). In contrast, *KCNN4*, which encodes a calcium activated potassium-ion channel, was the most downregulated gene in CH. Inhibition of KCNN4 expression in T lymphocytes has been shown to reduce T cell production of TNF-α and IFN-γ (26). Interestingly, the activation of KCNN4 by AP-1 has been shown to skew the differentiation of T helper cells towards a Th1/Th17 phenotype (27). The *FRMD8* gene product is critical for cytokine secretion, including TNF-α, of macrophages (28) and was significantly downregulated in UH PBMC suggesting the production of certain proinflammatory mediators is reduced in UH PBMC. In bison, *IL-6* and *TNFRSF4* expression was upregulated potentially impacting T cell responses. IL-6 is a multifunctional pro-inflammatory cytokine produced by lymphocytes and macrophages that is important for the initiation of systemic inflammatory responses, antibody production, and T-cell development (29). *TNFRSF4* encodes the OX40 receptor (CD134) which is involved in the clonal expansion and survival of CD4^+^ and CD8^+^ T cells often enhancing immune responses while conversely suppressing regulatory T cell activity (30).

Pathway analysis further revealed differences in the immune response between genotypes. The neutrophil degranulation pathway was significantly upregulated in Holsteins as compared to bison. Caseonecrotic lesions, comprised primarily of macrophages, neutrophils, and lymphocytes, are frequently observed in the lungs of *M. bovis* infected calves (31, 32). Neutrophil degranulation is important for elimination of pathogens though excessive degranulation can lead to immunopathology (33). Toll-like receptor 2 (TLR2) signaling is activated in response to *M. bovis* infection in bovine cells (34), promoting neutrophil degranulation and chemotaxis (35), and infection of bovine neutrophils increases elastase production (36). Further, *M. bovis* can degrade neutrophil extracellular traps (NETs) via expression of the membrane nuclease mnuA (37) reducing bacterial clearance and potentially promoting bacterial growth and increasing tissue damage (38). Upregulation of the neutrophil degranulation pathway in Holsteins, compared to bison, suggests differences in the host response.

Genotype and species differences in the adaptive immune response may be represented by the upregulation of the Class I MHC-mediated antigen processing and presentation pathway was upregulated in UH PBMC. Antigen presentation through the class I pathway activates CD8^+^ T cells and is essential for clearance of intracellular pathogens (39). As *M. bovis* has been shown to invade and persist in bovine turbinate cells (40) and macrophages (41, 42) an effective CD8^+^ T cell response could hypothetically resolve intracellular *M. bovis* infections. In contrast to Holsteins, bison had significant activation of the cachexia signaling pathway. Cachexia is a chronic wasting condition associated with loss of muscle and adipose tissue (43) from unresolved inflammation and prolonged pro-inflammatory cytokine expression, including IL-6, IL-10, IFN-γ, and TNF-α (44). This may correlate with disease pathogenesis in bison as loss of body condition is a frequently observed clinical sign in infected bison (9) and increased expression of genes involved in cachexia represent a putative molecular mechanism for disease pathogenesis in this species.

Pathogen induced cytokine storm is caused by immune dysregulation resulting in systemic inflammation affecting multiple organ systems associated with bacterial (45) and viral infections (46). Cytokine storms are associated with increased production of pro-inflammatory cytokines including IFN-γ, IL-1, IL-6, IL-10, IL-12, and TNF-α (47). We observed that bison PBMC significantly upregulated the expression of these pro-inflammatory cytokines following *M. bovis* infection while UH PBMC produced a more focused Th1-type response with upregulation of IFN-γ, IL-12, IL-22, CCL2, and CXCL10 while increased expression of IL-6, IL-10, and TNF-α were not observed. The Th1-type cytokine profile observed in *M. bovis* infected UH PBMC may hypothetically increase the clearance of *M. bovis* infected host cells thereby reducing bacterial reservoirs and dissemination. Further, the lack of IL-6, IL-10, and TNF-α upregulation suggests that UH are less likely to develop cytokine storms. In our study, CH PBMC did not upregulate cytokine gene expression despite previous studies showing bovine monocytes produce IL-10, but not IFN-γ or TNF-α, following *M. bovis* infection (48) which may be accounted for by methodological differences including the detection assay utilized, which measures the protein level rather than transcript, and a higher MOI. Future experiments, including a time-course comparing multiple MOI, may identify differential expression of *IL10*. The production of pro-inflammatory cytokines associated with cytokine storm by bison PBMC begins to provide mechanistic insight linking the host response to the severe clinical disease observed in *M. bovis* infected bison though further studies are required.

More broadly, Holstein PBMC upregulated the expression of several chemokine receptors, including CCR1, CCR5, CXCR3, and CXCR4, involved in leukocyte trafficking to sites of infection. CCR1 mediates T cell migration, by binding chemokines CCL3 and CCL5, to sites of infection and is critical for Th2 cytokine production (49); suggesting a mechanism for the observed Th2 cytokine profile in the lungs of *M. bovis* infected calves (50). CCR5 is a receptor for the chemokines CCL4 and CCL5 and has been shown to be important for the recruitment and maintenance of CD4^+^ T cells in the lungs of *Mycoplasma pulmonis* infected mice (51). CXCR3 binds three ligands, CXCL9, CXCL10, and CXCL11, and promotes migration of activated CD4^+^ and CD8^+^ T cells to infected tissue (52) though was also required for the accumulation of regulatory T cells at sites of inflammation (53). Bison and both Holstein genotypes upregulated CXCR4 expression in response to *M. bovis* infection. CXCR4 is expressed on the surface of proliferating cells (54) and has been shown to be upregulated on CD4^+^ T cells in children with severe *Mycoplasma pneumonia* infection (55). Interestingly, increased concentrations of the CXCR4 ligand, CXCL12, were observed in the lungs of *Mycoplasma pneumonia* infected mice (55). Further, inhibitor treatment downregulated the surface expression of CXCR4 reducing the concentrations of inflammatory cytokines (55) suggesting the CXCL12/CXCR4 axis is involved in the T cell response to *M. bovis* infection in large ruminants. Our data showed species-specific increases in the expression of multiple chemokine receptors, which have not been previously described in the host response to *M. bovis*. However, additional studies are required to further characterize chemokine receptor expression and their role in *M. bovis* disease pathogenesis.

Analysis of the PICSS pathway identified species-specific upregulation of DEG critical in the function of multiple programmed cell death pathways. Intriguingly, multiple genes required for inflammasome signaling, and initiation of programmed cell death pathways, show species-specific differential expression. Bison PBMC upregulated *TNFSF10*, *FADD, NLRP3*, and *MLKL* following *M. bovis* infection. *TNFSF10* encodes TNF-related apoptosis-inducing ligand (TRAIL) in activated PBMC inducing apoptosis (56) or, alternatively, necroptosis (57). NLRP3 inflammasome priming occurs through pathogen- and damage- associated molecular patterns receptors and requires FADD and caspase-8 binding to initiate programmed cell death pathways (58) and cytokine production (59). MLKL, mixed lineage kinase domain-like protein, mediates necroptosis through oligomerization and permeabilization of the plasma membrane (60). Stimulation of cells by TRAIL or by interferons (61) can induce MLKL mediated necroptosis in the absence of caspsase-8 (62), resulting in the release of damage associated molecular patterns (63). NLRP3 inflammasome activation can have protective or pathogenic effects during bacterial infection and it is currently unclear how NLRP3 functions during *M. bovis* infection in ruminants. Previous studies have shown NLRP3 activation increases the production of IL-1β and IL-6, the infiltration of myeloid cells, and is associated with increased tissue damage in the lungs of *Mycoplasma pneumoniae* infected mice (64, 65). Further studies will be required to elucidate species-specific activation of programmed cell death pathways and their contributions to bacterial clearance, disease resolution, and pathology.

Both genotypes of Holstein PBMC upregulated *CASP8* and *GSDME* in response to *M. bovis* infection. *CASP8* encodes for caspase-8, an initiator of apoptosis required for cleavage of subsequent caspases resulting in programmed cell death (66), and prevents the induction of necroptosis and associated immunopathology (67, 68). Additionally, caspase-8 has been shown to induce the synthesis of pro-IL-1β in *Salmonella Typhimurium* infected murine macrophages (69). *GSDME* encodes for gasdermin E, a pore forming protein, that has been shown to facilitate IL-1β release (70). Previous studies have demonstrated that Holstein PBMC produce IL-1β in response to stimulation with lipopolysaccharide and lipoteichoic acid, with greater production observed in UH PBMC (19, 71). Our study suggests IL-1β may be dependent on caspase-8 and gasdermin E functions though additional studies are required to elucidate species- and genotype-specific differences in IL-1β production.

The extensive selection of Holsteins for increased milk production concomitantly impacted CH immunogenetics thereby compromising responses to bacterial pathogens. Intensive selection for milk yield produced CH with decreased heterozygosity in major histocompatibility (MHC) regions, including class I bovine leukocyte antigen (BOLA)-A and class II BOLA-DRB2 and BOLA-DQA1 (13). A reduction in BOLA diversity could negatively impact antigen presentation potentially blunting the magnitude or breadth of T cell responses. IL-18R was affected by selection though functional effects on immune responses in Holsteins are currently unknown. IL-18R is expressed on myeloid and lymphoid cells where it induces pro-inflammatory cytokine production, including IFN-ϒ (72) though IL-18R signaling is linked to protective (73) or pathologic immune responses (74). Recent studies have highlighted the impact of Holstein selection on immunity to bacterial pathogens. Lippolis et al. (17) found UH to have lower milk somatic cell and *E. coli* counts following intramammary challenge compared to CH and Cousillas-Boam et al. (18) demonstrated increased serum TNF-α and IL-6 concentrations in UH after lipopolysaccharide treatment. Further, *ex vivo* stimulation of Holstein PBMC found increased production of IL-1β and IL-6 in UH (19, 71). These studies support the hypothesis that selection for increased milk yield compromised the immune response to bacterial infection in CH and suggest the UH genotype is immunologically more robust. Continued research, including *in vivo* challenge studies, is required to further identify the effects of genotype on bacterial infection outcomes in Holsteins.

Sexual dimorphism of the immune response influences outcomes following infection including the manifestation of clinical signs and immunopathology. In general, females mount stronger immune responses against infection compared to males, however, are more prone to develop inflammatory and autoimmune diseases (75). Dairy and bison herds are intensively managed and predominantly comprised of female animals (76) thus our study exclusively utilized PBMC from female Holsteins and bison. Bison cows predominantly exhibit clinical signs and have increased mortality rates compared to calves and adult bulls in affected herds (76–78) suggesting sexual dimorphism of the immune response potentially impacts *M. bovis* epidemiology and clinical outcomes. Estrogen receptors are expressed by myeloid and lymphoid cell types and can regulate the expression of TLR, cytokine, and chemokine genes, including IL-1, IL6, and IL-10 (79–81). Females have greater numbers of CD4^+^ T cells that produce significantly greater levels of TNF, IL-6, IL-17, and IFN-ϒ. In cattle, heifers produced greater concentrations of TNF-α, compared to bulls, following lipopolysaccharide challenge despite bulls exhibiting more severe clinical signs (82). Further, a bovine herpesvirus-1, *Mannheimia haemolytica* coinfection increased circulating leukocytes in heifers compared to bulls (83). The data presented herein may have resulted in the activation of different gene signaling pathways, particularly the PICCS and cachexia pathway, had bull PBMC been tested. These findings suggest cows, in particular bison cows, may be at higher risk for complications following *M. bovis* infection.

Holsteins and bison display observable differences in *M. bovis* clinical disease and epidemiology. Though the respiratory disease caused by *M. bovis* is variable, in cattle *M. bovis* is primarily associated with polymicrobial BRD causing chronic bronchopneumonia, polyarthritis, and otitis media mainly in calves and yearlings (84, 85). *M. bovis* is a primary pathogen of bison causing caseonecrotic pneumonia and disseminated, systemic disease with lethargy and loss of body condition common in moribund animals (7, 8, 86). In contrast to cattle, clinical signs are largely absent in bison calves while most clinical signs and mortalities are observed in adult females (78, 86), though recent data suggest bulls may be equally affected (76). Our results begin to provide context for the species-specific clinical signs and epidemiology. Upregulation of genes involved in cachexia and PICSS suggest these factors may contribute to the lethargy and loss of body condition observed in bison, though further research is required to comprehensively elucidate the molecular mechanisms contributing to *M. bovis* disease and immunopathology in large ruminants. Additionally, future studies comparing the transcriptomic response of male and female bison to *M. bovis* infection will provide context on sex as a factor for severe disease in bison.

Development of novel vaccines is imperative to control *M. bovis* in bison. Vaccination studies have shown markedly differing responses to experimental vaccines between Holsteins and bison. Briggs et al. (87) demonstrated intranasal vaccination with a modified-live *Mannheimia haemolytica* expressing the *M. bovis* antigens EF-Tu and Hsp70 successfully reduced lung bacterial loads in Holstein calves while a similar study evaluating the same vaccine in bison reported no differences in lung bacterial loads or pathology (88) demonstrating the dichotomy of responses between Holsteins and bison. Recently, we reported that an injectable subunit vaccine comprised of recombinant *M. bovis* Elongation Factor Tu and Heat Shock Protein 70 reduced lung bacterial loads and lung lesion formation in vaccinate bison (89). Transcriptomic analysis of serially collected blood samples found upregulation of TBX21, required for type I immunity, and PAX5, a transcription factor required for B cell differentiation and significant enrichment of pathways involved in T cell differentiation, cell adhesion, viral infection, and the adaptive immune response (90). Following challenge, increased expression of genes comprising modules for Fcϒ receptor mediated phagocytosis, NK cell cytotoxicity, and lysosome activity were observed. These data show the adjuvanted protein subunit vaccine promotes Th1 type immune response, hypothesized to be beneficial for *M. bovis* infection in large ruminants, in the absence of upregulation of pathways associated with immunopathology, including PICSS and cachexia.

*M. bovis* is a unique bacterial pathogen of cattle and bison that severely impacts animal health and causes significant economic losses. Despite the detrimental impact of *M. bovis* on the health of cattle and bison, effective intervention strategies for *M. bovis*, particularly in bison, are lacking. The current study represents the first comparison of CH, UH, and bison PBMC transcriptomic responses following *M. bovis* infection. These data further support the hypothesis that intensive selection of Holsteins for increased milk-production during the 1960’s, concurrent with the emergence of *M. bovis*, altered the immune response to bacterial pathogens. Additionally, this study demonstrates the host response of bison to *M. bovis* infection differs from that of Holsteins with the gene expression profiles providing initial mechanistic evidence for the differences in clinical disease and epidemiology observed between bison and cattle. Further comparisons of host responses of susceptible species will provide increased understanding of molecular mechanisms underlying the dichotomy of clinical maladies and may be beneficial in the development of novel intervention strategies for both species.

## Data Availability

The datasets presented in this study can be found in online repositories. The names of the repository/repositories and accession number(s) can be found below: https://www.ncbi.nlm.nih.gov/genbank/, PRJNA 1439942.
